# Fibrinogen alpha C chain 5.9 kDa fragment (FIC5.9), a biomarker for various pathological conditions, is produced in post-blood collection by fibrinolysis and coagulation factors

**DOI:** 10.1186/s12014-016-9129-6

**Published:** 2016-10-07

**Authors:** Wataru Kikuchi, Motoi Nishimura, Takahisa Kuga, Sachio Tsuchida, Tatsuya Saito, Mamoru Satoh, Kenta Noda, Yoshio Kodera, Takeshi Tomonaga, Fumio Nomura

**Affiliations:** 1 Department of Molecular Diagnosis, Graduate School of Medicine, Chiba University, Chiba, Japan; 2 R&D Department, Nittobo Medical Co., Ltd., Koriyama, Japan; 3 Department of Biochemistry and Molecular Biology, Kyoto Pharmaceutical University, Kyoto, Japan; 4 Department of Physics, School of Science, Kitasato University, Sagamihara, Kanagawa Japan; 5 Laboratory of Proteome Research, National Institute of Biomedical Innovation, Osaka, Japan; 6 Division of Clinical Mass Spectrometry and Clinical Genetics, Chiba University Hospital, Chiba, Japan

**Keywords:** Biomarker, Fibrinogen, Plasmin, Thrombin, Fibrinogen alpha C chain 5.9 kDa fragment (FIC5.9), Coagulation, Hepatitis

## Abstract

**Background:**

Fibrinogen alpha C chain 5.9 kDa fragment (FIC5.9) is a new serum biomarker for chronic hepatitis that was discovered by proteomics analysis. Previous studies have shown that FIC5.9 is derived from the C-terminal region of fibrinogen alpha chain and the serum levels of FIC5.9 decrease in chronic hepatitis. It also have been reported that FIC5.9 cannot be detected in the blood stream of the systemic circulation and it is released from fibrinogen during blood clotting in collecting tube. However, the mechanism of FIC5.9 releasing from fibrinogen is unclear.

**Methods:**

We formulated a hypothesis that FIC5.9 is released by enzymes that are activated by post-blood collection and may be coagulation and fibrinolysis factors. In this study, we analyzed the mechanisms of FIC5.9 releasing from fibrinogen in healthy blood.

**Results:**

Our analysis showed that thrombin acts as an initiator for FIC5.9 releasing, and that mainly plasmin cleaves N-terminal end of FIC5.9 and neutrophil elastase cleave C-terminal end of FIC5.9.

**Conclusion:**

FIC5.9 reflects minute changes in coagulation and fibrinolysis factors and may be associated with pathological conditions.

**Electronic supplementary material:**

The online version of this article (doi:10.1186/s12014-016-9129-6) contains supplementary material, which is available to authorized users.

## Background

Many biomarkers have been discovered by proteomics analysis, but fewer have been developed for clinical use [1, 2]. Most of the reported biomarkers involve posttranslational modification or degradation, and they are unclear why the level of biomarker changes in disease. Thus, there is a need to establish the links between synthetic mechanism of the biomarker and disease conditions for practical use in clinical diagnosis [3].

**Fig. 1:**
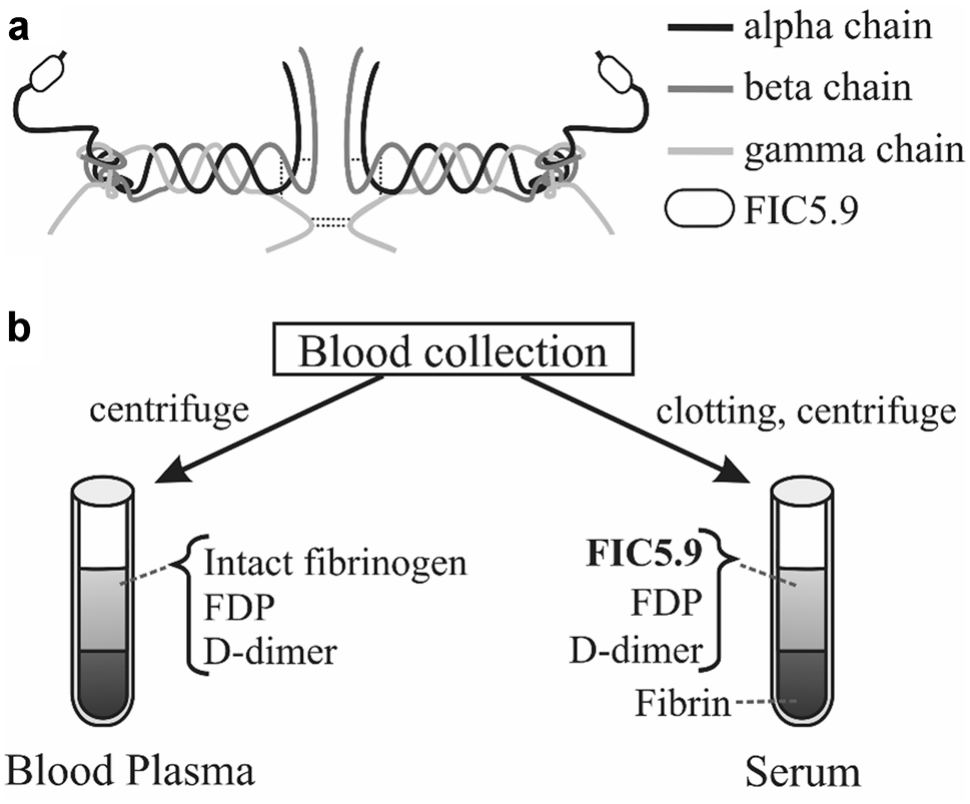
Fibrinogen and FIC5.9 region (**a**). A model of FIC5.9 synthesis (**b**). FIC5.9 is released from fibrinogen during the process of blood coagulation [8]. FIC5.9 is not found in blood plasma and only detected in serum after blood clotting

Fibrinogen alpha C chain 5.9 kDa fragment (FIC5.9) is a new serum marker for chronic hepatitis that was discovered in samples from alcoholic liver disease using SELDI-TOF MS [4]. FIC5.9 is derived from the C-terminal region of fibrinogen alpha chain and its molecular weight is 5890 Da (Fig. 1a). It has been established that the level of FIC5.9 is high in healthy people and low with onset of hepatitis [5–7]. We developed an ELISA system for FIC5.9 and evaluated the clinical utility of FIC5.9 in various kinds of chronic hepatitis [8]. The results showed that the serum level of FIC5.9 is significantly decreased in the early stage of liver fibrosis and showed a strong indicator for liver fibrosis. We also found that natural FIC5.9 releasing from fibrinogen is negligible in the blood circulation of healthy subjects, but clearly detected in the serum collection tubes, thus we concluded that FIC5.9 is released from fibrinogen during the process of blood coagulation [8]. That is, FIC5.9 is an in vitro product that is released during blood clotting from fibrinogen (Fig. 1b). Since our initial identification of FIC5.9, the same peptide (if not identified completely, but with the completely same mass) has been reported as a biomarker in several pathological conditions, including cancers and inflammatory disorders [9–14]. However, the mechanism of FIC5.9 releasing from fibrinogen is unknown.

**Fig. 2:**
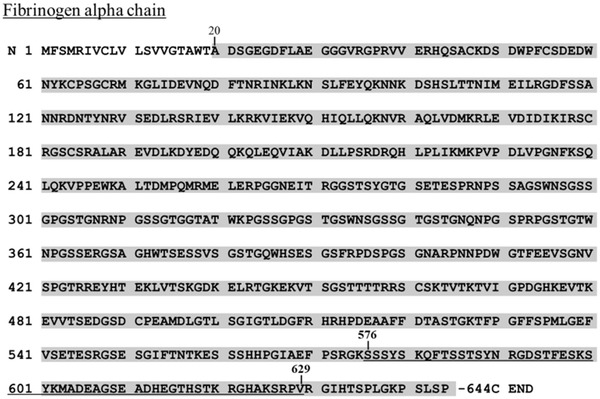
Sequences of fibrinogen alpha chain and FIC5.9. The mature alpha chain is indicated by a *grey shadow*. The FIC5.9 sequence (amino acids 576–629) is *underlined*

Based on the studies above, we formulated a hypothesis that FIC5.9 is released from fibrinogen or fibrin by the enzyme that is activated post-blood collection. Especially we focused on the coagulation and fibrinolysis factors. FIC5.9 sequence includes amino acids 576–629 of the fibrinogen alpha chain (Fig. 2) and FIC5.9 appears to be synthesized by cleavage at RGK/SSS (N-terminal region) and RPV/RGI (C-terminal region) [4]. Coagulation and fibrinolysis factor, thrombin and plasmin are major enzymes that cleave to the carboxyl side of lysine work with fibrinogen or fibrin [15]. There are fewer reports of enzymes in blood that cleave to the carboxyl side of valine, but neutrophil elastase is one of such example [16]. In this study, as a first step toward understanding the mechanisms of FIC5.9 changes in various pathologies, we tested the hypothesis that FIC5.9 is released by these enzymes in apparently healthy subjects.

## Methods

### FIC5.9 synthesis during blood clotting

To confirm the conclusion of our previous study [8] regarding the roles of coagulation factors in synthesis of FIC5.9, the coagulation cascade was reactivated by adding Thrombocheck APTT-SLA (Sysmex Corp., Hyogo, Japan) to coagulation-deficient plasma (factor II, V, VII, VIII, IX, X, XI, XII; Sysmex). Control plasma was collected with Insepack II (sodium citrate type, Sekisui Medical Co., Tokyo, Japan). After 1-h incubation at 25 °C, samples were centrifuged at 1500*g* for 20 min. The supernatant was purified with C18/WCX cartridges [4]. FIC5.9 levels in samples were measured by MALDI-TOF MS on a Bruker AUTOFlex^®^ mass spectrometer, using stable isotope-labeled FIC5.9 as an internal standard [4]. Each experiment was done in triplicate.

### In vitro degradation of purified fibrinogen

Purified fibrinogen (Wako Pure Chemical Industries, Tokyo, Japan; 70 µg) in PBS buffer was incubated with thrombin (Wako; final conc. 2 U/mL [17]), plasmin (Wako; 1 U/mL [18]) or neutrophil elastase (Sigma-Aldrich, St. Louis, MO, USA; 1 U/mL [19]) for 2 h at 25 °C. The reaction was stopped by adding EDTA (pH 8.0, 10 mM) and aprotinin (Wako; 1 U/mL). Under these conditions, purified fibrinogen is extensively degraded by thrombin, plasmin and neutrophil elastase [17–19].

### LC–MS/MS analysis of degradation products of fibrinogen

StageTips C18 (Thermo Fisher Scientific, Waltham, MA, USA) was used for desalting the degradation products of fibrinogen [20]. Obtained peptides were identified by LC/MS/MS analysis [21].

### Analysis and time course of FIC5.9 synthesis in serum collection tubes

Serum collection tubes (tubes are evacuated; Insepack II, Sekisui Medical Co.) with added thrombin (Wako; 20 U/mL), hirudin (Thermo Fisher; 1 U/mL [17]), plasmin (Wako; 0.8 U/mL), tranexamic acid (Wako; 10 mM [18]), sivelestat sodium (Cosmo Bio Co.; Tokyo Japan; 80 µM [19]) or the same volume of saline were used to collect blood samples from eleven healthy volunteers with an evacuated by Safetouch™ Blood Collection system (NIPRO Co., Osaka Japan). The collected blood was clotted for 0, 5, 30, 60, 90 min at 25 °C. After blood clotting, serum was obtained by centrifugation at 1500*g* for 10 min at 4 °C. The level of FIC5.9 was measured using a FIC5.9 ELISA kit described in [6]. Written informed consent was obtained prior to the sample collection, and the study was approved by the research ethics committee of the graduate school of medicine, Chiba University (Approval no. 677).

### Statistical analysis

Statistical analysis was performed by Mann–Whitney U-test with SPSS software, version 18.0 (SPSS Inc., Chicago, IL, USA). A P value <0.05 were considered significant.

## Results

### LC–MS/MS analysis of degradation products of fibrinogen in vitro

**Fig. 3:**
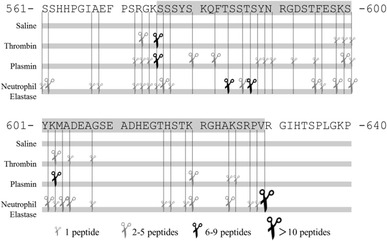
Cleavage site mapping of thrombin, plasmin and neutrophil elastase in FIC5.9 surrounding regions. Cleavage sites are indicated with scissors, depending on the number of peptides identified by LC–MS/MS analysis. Identified peptide sequence information is shown in Additional file 2

To reconfirm that FIC5.9 is released from fibrinogen in the serum collection tube during blood clotting [8], we studied FIC5.9 releasing analysis using coagulation-deficient plasma. Deficiency of factor II, V, VIII and X decreased the FIC5.9 releasing from fibrinogen (Additional file 1), and that factor II (thrombin) had a particularly marked effect on the releasing of FIC5.9 from fibrinogen. We reconfirmed that FIC5.9 is released in the serum collection tube during blood clotting. To determine the coagulation and fibrinolysis factors responsible to release FIC5.9, we narrowed down the candidate enzymes based on these results. Information of enzyme cleavage sites was obtained from the Peptidase Database (MEROPS: http://merops.sanger.ac.uk. Accessed 21 September 2013). and we finally choose thrombin, plasmin, and neutrophil elastase [17–19]. We degraded purified fibrinogen with these enzymes and the sequences of degradation products were analyzed by LC–MS/MS (Fig. 3; Additional file 2). Our degradation conditions showed that several cleavage sites are found in fibrinogen alpha C terminal domain, but especially the N-terminal region of FIC5.9 (RGK/SSS) was cleaved by thrombin or plasmin, and the C-terminal region of FIC5.9 (RPV/RGI) was cleaved by neutrophil elastase. While we tried fibrinogen digestion experiment, using thrombin, plasmin, neutrophil elastase, and their mixture, we could not form FIC5.9 itself from fibrinogen, but observed its fragments in vitro (data not shown).

### Analysis and time course of FIC5.9 releasing in serum collection tubes

To determine if thrombin, plasmin and neutrophil elastase can release FIC5.9 from fibrinogen in blood, we analyzed the time course of FIC5.9 releasing in an evacuated blood collection system. We firstly examined the time course of FIC5.9 releasing in plain and silica-coated tubes by measuring the levels of FIC5.9 in serum after 0, 5, 30, 60, 90 min of clotting. The rate of FIC5.9 releasing in a silica-coated tube was significantly faster than that in a plain tube (Additional file 3), but the final FIC5.9 levels did not differ significantly. All further analyses were performed in silica-coated tubes.

**Fig. 4:**
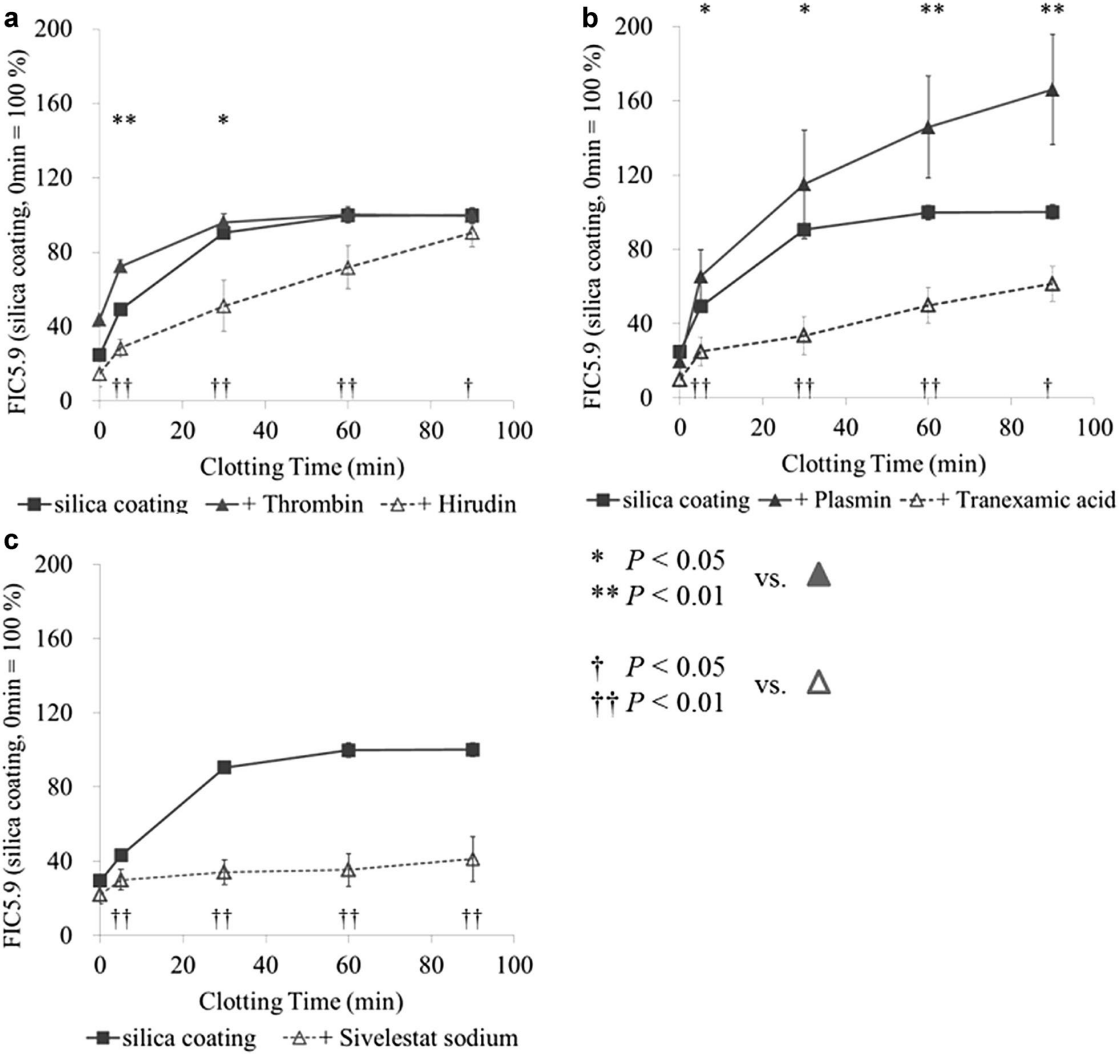
Analysis of time course of FIC5.9 releasing with thrombin spiked/inhibited (**a**), plasmin spiked/inhibited (**b**), or neutrophil elastase inhibited (**c**) serum collection tubes. The relative amount of FIC5.9 was measured by FIC5.9 ELISA. Statistical comparison of silica-coated tube versus enzyme-spiked tube (*asterisk*), or inhibitor spiked tube (*dagger symbol*) was performed as described in the “Methods” section

In the next, we examined the time course of FIC5.9 releasing containing thrombin, hirudin (a thrombin inhibitor), plasmin, tranexamic acid (a plasmin inhibitor), or sivelestat sodium (a neutrophil elastase inhibitor) (Fig. 4a–c). In all experiments (Fig. 4), 0 min of clotting could not eliminate a time lag of serum separation (a time for blood reaches from venous blood collection needle to blood collection tube, and centrifugation duration), and FIC5.9 level was not found to be zero at 0 min. The rate of FIC5.9 releasing from fibrinogen with added thrombin was significantly faster than that in silica-coated tubes. FIC5.9 releasing with added thrombin reached a plateau after 30 min clotting, but the amount of FIC5.9 was same with silica-coated and plain tubes (Fig. 4a). Addition of hirudin (thrombin inhibitor) significantly delayed the FIC5.9 releasing, but prolonged to after 90 min of clotting. Addition of plasmin significantly increased the releasing rate and total amount of FIC5.9, and addition of tranexamic acid (plasmin inhibitor) markedly decreased the FIC5.9 releasing (Fig. 4b). Addition of sivelestat sodium (neutrophil elastase inhibitor) strongly inhibited FIC5.9 releasing (Fig. 4c).

## Discussion

In this study, we formulated a hypothesis that FIC5.9 is released from fibrinogen by coagulation and fibrinolysis factors. We firstly analyzed in vitro released fragments from fibrinogen by digestion with thrombin, plasmin or neutrophil elastase using LC–MS/MS (Fig. 3). Successively we analyzed the time course of FIC5.9 release was determined in serum collection tubes spiked with thrombin (and its inhibitor, hirudin), plasmin (and its inhibitor, tranexamic acid) and sivelestat sodium (neutrophil elastase inhibitor) (Fig. 4). We concluded our analysis showed that thrombin acts as an initiator for FIC5.9 releasing, and that mainly plasmin cleaves N-terminal end of FIC5.9 and neutrophil elastase cleave C-terminal end of FIC5.9 from fibrinogen.

LC–MS/MS analysis showed that N-terminal end of FIC5.9 (RGK/SSS) is cleaved by thrombin or plasmin, and C-terminal end of FIC5.9 (RPV/RGI) is cleaved by neutrophil elastase (Fig. 3). To confirm that these enzymes can release FIC5.9 during blood coagulation, we analyzed the time course of FIC5.9 release was determined in serum collection tubes spiked with thrombin (and its inhibitor, hirudin), plasmin (and its inhibitor, tranexamic acid) and sivelestat sodium (neutrophil elastase inhibitor) (Fig. 4). Addition of thrombin (or their inhibitor, hirudin) accelerated (or delayed) the releasing of FIC5.9 from fibrinogen, but the amounts of FIC5.9 at 90 min clotting had no significance. It is clear that blood clotting initiates FIC5.9 releasing, and FIC5.9 is negligible in the blood circulation, but found in serum collection tubes. However, these results do not demonstrate thrombin plays a main role in FIC5.9 releasing when once blood clotting process is started in serum collection tubes. We conclude thrombin play a role of initiation element of FIC5.9 releasing by starting blood clotting with its activity.

On the other hands, addition of plasmin sufficiently accelerated the releasing of FIC5.9 and increased substantially the amount of FIC5.9. While thinking thrombin is an initiation element of FIC5.9 releasing, these results indicate that plasmin is the major enzyme that cleaves the N-terminal region of FIC5.9 and affects the amount of FIC5.9. In nature, activation of plasminogen to plasmin occurs after activation of coagulation factors including thrombin [22, 23]. These reports also support the role of thrombin as the initiator of FIC5.9 synthesis. Inhibition of plasmin activity significantly suppressed FIC5.9 releasing from fibrinogen (Fig. 4). The suppression of plasmin by tranexamic acid was significant, but not completely. It may be due to other enzymes which can cleave the N-terminal end of FIC5.9; in fact, our in vitro digestion experiments (Fig. 3) show thrombin can cleave N-terminal end of FIC5.9. Taken together, plasmin seems to play a main role in cleaving N-terminal end of FIC5.9 and releasing FIC5.9.

Up to here, we discussed enzymes cleaving N-terminal end of FIC5.9. Our results also indicate neutrophil elastase play an important role in cleaving C-terminal end of FIC5.9 (Figs. 3, 4). Results of the in vitro digestion experiments (Fig. 3) showed that C-terminal end of FIC5.9 (RPV/RGI) is cleaved by neutrophil elastase, while inhibition of its activity strongly suppress FIC5.9 releasing in serum collection tube (Fig. 4). The suppression of neutrophil elastase completely inhibited the releasing of FIC5.9 from fibrinogen (Fig. 4c), which implies neutrophil elastase plays a major role in cleaving the C-terminal end and releasing of FIC5.9 from fibrinogen.

In the experiments of serum collection tubes, FIC5.9 was detected at 0 min of clotting or inhibitor added tubes (Fig. 4). We estimate this phenomenon is caused by blood collection process; by the moment blood reaches serum collection tubes, blood passes blood collection needle and a thin tube connecting between the needle and a serum collection tube, clearly blood coagulation process have already started before blood reaches serum collection tubes; and centrifugation duration is 10 min.

Our results showed that the mechanism of FIC5.9 releasing in healthy people is strongly related to coagulation and fibrinolysis factors. And our results suggest why FIC5.9 is a marker of alcoholic liver disease and liver fibrosis [4–8]. Neutrophil elastase cleaves the C-terminal site of FIC5.9, but only a few reports indicate that the level of neutrophil elastase (or the neutrophil count) changes in the early stage of chronic hepatitis [24, 25]. Therefore, the C-terminal site of FIC5.9 is likely to be cleaved at a similar level in healthy people and patients with chronic hepatitis. However, an extreme increase or decrease in neutrophils might affect FIC5.9 releasing. A spike test of neutrophil elastase into the collection tube could not be performed because we could not obtain enough amount of elastase. Indeed, significant volume of enzyme was needed for experiment with blood collection tubes. However, the results of LC–MS/MS indicated that neutrophil elastase can cleave the C-terminal region of FIC5.9. This requires confirmation in a further study.

Thrombin (seems to be an initiator of FIC5.9 synthesis) and plasmin (which significantly cleaves the N-terminal site of FIC5.9) are molecular markers of liver disease, partly because both enzymes are secreted from liver [26–29].

The presence of other factors related to FIC5.9 releasing should also be considered. Recently, Marfa et al. reported that TGF-β reduces the expression level of fibrinogen alpha chain mRNA [7], which is of note because the level of TGF-β is related to liver fibrosis and hepatitis [31, 32]. In addition, plasma fibrinogen has been proposed as a marker for chronic liver disease [33]. As these reports show, the low level of FIC5.9 releasing in chronic hepatitis is not simply due to enzymatic reactions, but also to a decrease in fibrinogen synthesis (a decrease in the precursor to FIC5.9). Thus, it seems that several factors are involved in determining the level of FIC5.9.

## Conclusion

Measurement of degradation products in blood circulation is commonly used in clinical tests [34, 35]. Most coagulation and fibrinolysis factors are unstable for measurement of activity [36–38], as exemplified by the activated partial thromboplastin time (APTT) and the prothrombin time (PT). FIC5.9 is also a degradation product from fibrinogen alpha chain that is released by coagulation and fibrinolysis factors, and reflects a minute change in these factors. Thrombin, plasmin and neutrophil elastase are involved in the key mechanism of FIC5.9 releasing in clotting of normal blood. This provides the basis for understanding the decrease in FIC5.9 in clotting of blood from patients with chronic hepatitis. Further analysis may show similar effects in blood from patients with several diseases.

## Additional files

10.1186/s12014-016-9129-6 Analysis of FIC5.9 releasing in coagulation factor-deficient plasma. (A) Mass spectrum of coagulation-depleted plasma reactivated using an APTT reagent. Synthesized FIC5.9 is indicated with a red line and SI-labeled FIC5.9 peptide is indicated with a blue line. (B) Quantification of FIC5.9 released by coagulation reactivation. The relative intensity of FIC5.9 was calculated by comparison with the intensity of the internal standard (SI-FIC5.9). The error bars represent the standard error of the mean (SEM) for three experiments.

10.1186/s12014-016-9129-6 Peptides cleaved by thrombin, plasmin and neutrophil elastase in FIC5.9 and surrounding regions identified by LC-MS/MS analysis.

10.1186/s12014-016-9129-6 Analysis of synthesis and time course of FIC5.9 in plain and silica-coated tubes. The relative amount of FIC5.9 was measured by FIC5.9 ELISA. Statistical analysis was performed as described in the Methods.

## Abbreviations

FIC5.9: fibrinogen alpha C chain 5.9 kDa fragment
MS: mass-spectrometry
SELDI-TOF MS: surface-enhanced laser desorption/ionization-time of flight mass spectrometry
ELISA: enzyme-linked immunosorbent assay
MALDI-TOF MS: matrix assisted laser desorption/ionization-time of flight mass spectrometry
APTT: activated partial thromboplastin time
