# Supplementary figures and images for: Fibrinogen alpha C chain 5.9 kDa fragment (FIC5.9), a biomarker for various pathological conditions, is produced in post-blood collection by fibrinolysis and coagulation factors
Source: Clin Proteomics. 2016 Oct 7;13:27. doi: 10.1186/s12014-016-9129-6 (PMC5055723; doi:10.1186/s12014-016-9129-6)

**S1 Fig.**


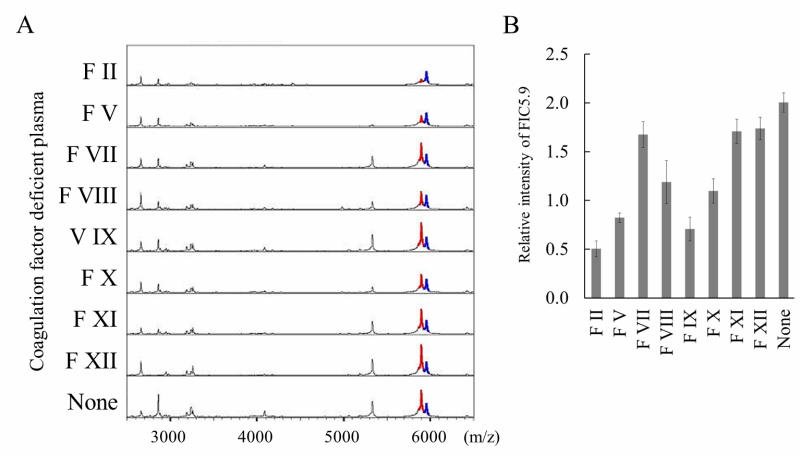

Supplement: Supplementary file 1 — 10.1186/s12014-016-9129-6 Analysis of FIC5.9 releasing in coagulation factor-deficient plasma. (A) Mass spectrum of coagulation-depleted plasma reactivated using an APTT reagent. Synthesized FIC5.9 is indicated with a red line and SI-labeled FIC5.9 peptide is indicated with a blue line. (B) Quantification of FIC5.9 released by coagulation reactivation. The relative intensity of FIC5.9 was calculated by comparison with the intensity of the internal standard (SI-FIC5.9). The error bars represent the standard error of the mean (SEM) for three experiments. [file 12014_2016_9129_MOESM1_ESM.docx]

**S2 Fig**


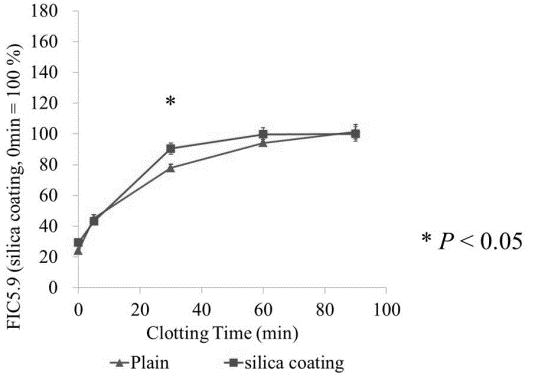

Supplement: Supplementary file 3 — 10.1186/s12014-016-9129-6 Analysis of synthesis and time course of FIC5.9 in plain and silica-coated tubes. The relative amount of FIC5.9 was measured by FIC5.9 ELISA. Statistical analysis was performed as described in the Methods. [file 12014_2016_9129_MOESM3_ESM.docx]
