# Supplementary material for: Fibrinogen alpha C chain 5.9 kDa fragment (FIC5.9), a biomarker for various pathological conditions, is produced in post-blood collection by fibrinolysis and coagulation factors
Source: Clin Proteomics. 2016 Oct 7;13:27. doi: 10.1186/s12014-016-9129-6 (PMC5055723; doi:10.1186/s12014-016-9129-6)
Supplement: Supplementary file 2 — 10.1186/s12014-016-9129-6 Peptides cleaved by thrombin, plasmin and neutrophil elastase in FIC5.9 and surrounding regions identified by LC-MS/MS analysis. [file 12014_2016_9129_MOESM2_ESM.docx]

**S1 Table.** Peptides cleaved by thrombin, plasmin and neutrophil elastase in FIC5.9 and surrounding regions identified by LC-MS/MS analysis.

| Enzyme | Confirmed sequences |
| --- | --- |
| Thrombin  (7 Peptides) | 574- GKSSSYSKQFTSSTSYNRGDSTFESKSYKMADEA -604 |
|  | 574- GKSSSYSKQFTSSTSYNRGDSTFESKSYKM -603 |
|  | 576- SSSYSKQFTSSTSYNRGDSTFESKSYKMA -604 |
|  | 576- SSSYSKQFTSSTSYNRGDSTFESKSYKM -603 |
|  | 576- SSSYSKQFTSSTSYNRGDSTFESKS -600 |
|  | 576- SSSYSKQFTSSTSYNRGDSTFESK -599 |
|  | 576- SSSYSKQFTSSTSYNRGDSTFES -598 |
| Plasmin  (26 Peptides) | 528- FPGFFSPMLGEFVSETESRGSESGIFTNTKESSSHHPGIAEFPSRGK -575 |
|  | 528- TFPGFFSPMLGEFVSETESRGSESGIFTNTKESSSHHPGIAEFPSRG -574 |
|  | 528- TFPGFFSPMLGEFVSETESRGSESGIFTNTKESSSHHPGIAEFPSR -573 |
|  | 528- TFPGFFSPMLGEFVSETESRGSESGIFTNTKESSSHHPGIAEFPS -572 |
|  | 540- FVSETESRGSESGIFTNTKESSSHHPGIAEFPSRGK -575 |
|  | 541- VSETESRGSESGIFTNTKESSSHHPGIAEFPSRGK -575 |
|  | 548- GSESGIFTNTKESSSHHPGIAEFPSRGK -575 |
|  | 548- GSESGIFTNTKESSSHHPGIAEFPSRGKSSSYS -580 |
|  | 559- ESRGSESGIFTNTKESSSHHPGIAEFPSRGKSSSYSK -582 |
|  | 559- ESRGSESGIFTNTKESSSHHPGIAEFPSRGK -575 |
|  | 559- ESRGSESGIFTNTKESSSHHPGIAEFPSRG -574 |
|  | 559- ESSSHHPGIAEFPSRGKSSSYS -580 |
|  | 576- SSSYSKQFTSSTSYNRGDSTFESKSYK -602 |
|  | 576- SSSYSKQFTSSTSYNRGDSTFESKS -600 |
|  | 576- SSSYSKQFTSSTSYNRGDSTFESK -599 |
|  | 576- SSSYSKQFTSSTSYNRGDSTFES -598 |
|  | 576- SSSYSKQFTSSTSYNRGDST -595 |
|  | 576- SSSYSKQFTSSTSYNRG -592 |
|  | 576- SSSYSKQFTSSTSYN -590 |
|  | 576- SSSYSKQFTSSTSY -589 |
|  | 576- SSSYSKQFTSSTS -588 |
|  | 582- QFTSSTSYNRGDSTFESKSYK -602 |
|  | 582- QFTSSTSYNRGDSTFESKS -600 |
|  | 603- MADEAGSEADHEGTHSTKRGHAK -625 |
|  | 603- MADEAGSEADHEGTHSTKRGHA -624 |
|  | 603- MADEAGSEADHEGTHSTK -620 |
| Neutrophil  Elastase  (39 peptides) | 557- NTKESSSHHPGIAEFPSRGKSS -577 |
|  | 557- NTKESSSHHPGIAEFPSRGKS -576 |
|  | 557- NTKESSSHHPGIAEFPSRGK -575 |
|  | 558- TKESSHHPGIAEFPS -572 |
|  | 561- SSHHPGIAEFPSRGKS -576 |
|  | 562- SHHPGIAEFPSRG -574 |
|  | 562- SHHPGIAEFPSR -573 |
|  | 568- AEFPSRGKSSSYSKQFT -584 |
|  | 569- EFPSRGKSSSYSKQFT -584 |
|  | 585- SSTSYNRGDSTFESKSYKMADEAGSEADHEGTHSTKRGHAKSRPV -629 |
|  | 585- SSTSYNRGDSTFESKSYKMADEAGSEADHEGTHSTK -620 |
|  | 585- SSTSYNRGDSTFESKSYKMA -604 |
|  | 585- SSTSYNRGDSTFESKSYKM -603 |
|  | 585- TSYNRGDSTFESKSYKMADEAGSEADHEGTHSTKRGHAKSRPV -629 |
|  | 587- TSYNRGDSTFESKSYKMA -604 |
|  | 587- TSYNRGDSTFESKSYKM -603 |
|  | 588- SYNRGDSTFESKSYKMADEAGSEADHEGTHSTKRGHAKSRPV -629 |
|  | 588- SYNRGDSTFESKSYKMADEAGSEADHEGTHSTKRGHAKSRPV -629(O) |
|  | 588- SYNRGDSTFESKSYKMADEAGSEADHEGTHSTKRGHAK -625 |
|  | 588- SYNRGDSTFESKSYKMADEAGSEADHEGTHSTK -620 |
|  | 588- SYNRGDSTFESKSYKMADEAGSEADHEGTH -617 |
|  | 588- SYNRGDSTFESKSYKMADEA -607 |
|  | 588- SYNRGDSTFESKSYKMA -604 |
|  | 588- SYNRGDSTFESKSYKM -603 |
|  | 588- SYNRGDSTFESKSYK -602 |
|  | 596- FESKSYKMADEAGSEADHEGTHSTKRGHAKSRPV -629 |
|  | 596- FESKSYKMADEAGSEADHEGTHSTKRGHAK -625 |
|  | 597- ESKSYKMADEAGSEADHEGTHSTKRGHAKSRPV -629 |
|  | 599- KSYKMADEAGSEADHEGTHSTKRGHAKSRPV -629 |
|  | 599- KSYKMADEAGSEADHEGTHSTKRGHAKSRPV -629(O) |
|  | 599- KSYKMADEAGSEADHEGTHSTKRGHAKSR -627 |
|  | 599- KSYKMADEAGSEADHEGTHSTK -620 |
|  | 599- KSYKMADEAGSEADHEGTHST -619 |
|  | 599- KSYKMADEAGSEADHEGT -616 |
|  | 601- YKMADEAGSEADHEGTHSTKRGHAKSRPV -629 |
|  | 601- YKMADEAGSEADHEGTHSTKRGHAKSRPV -629(O) |
|  | 601- YKMADEAGSEADHEGTHSTKRGHAKSRP -628 |
|  | 601- YKMADEAGSEADHEGTHSTKRGHAK -625 |
|  | 603- ADEAGSEADHEGTHSTKRGHAKSRPV -629 |
| None | N.D. |

(O) indicates “oxidized”, and duplication is eliminated.
